# Supplementary material for: Role of RadA and DNA Polymerases in Recombination-Associated DNA Synthesis in Hyperthermophilic Archaea
Source: Biomolecules. 2020 Jul 14;10(7):1045. doi: 10.3390/biom10071045 (PMC7407445; doi:10.3390/biom10071045)
Supplement: Supplementary file 1 [file biomolecules-10-01045-s001.pdf]

SUPPLEMENTAL DATA

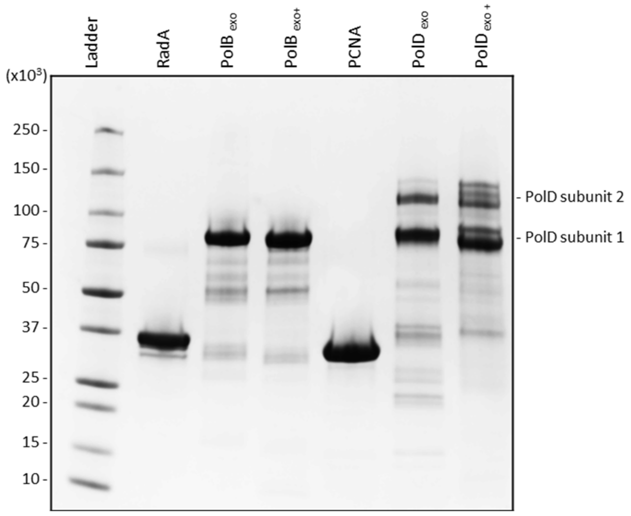

Figure S1. Electrophoretic profile of purified *P. abyssi* proteins.
3 µg of recombinant protein were loaded after denaturation in SDS-PAGE polyacrylamide gel. Ladder = Precision plus protein standards (BioRad).

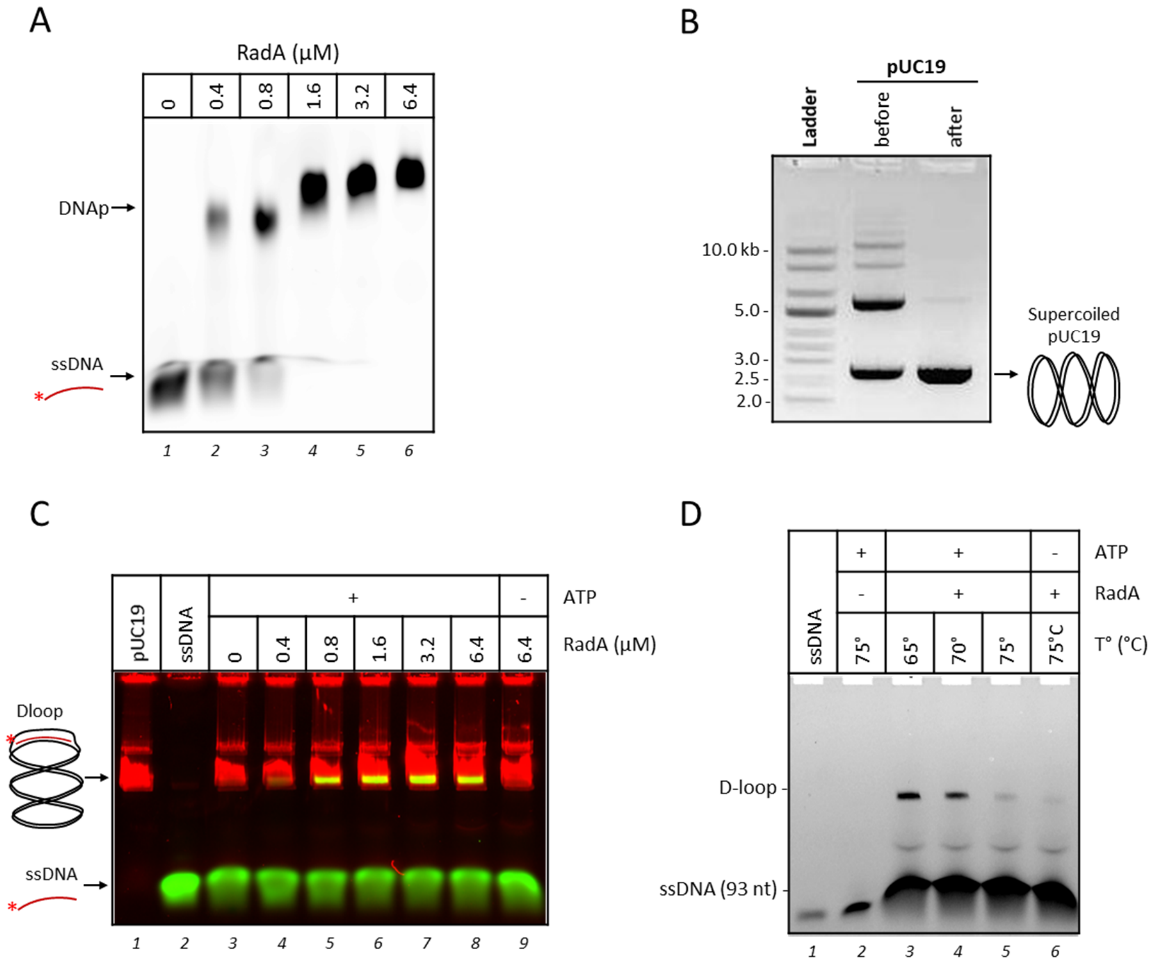

Figure S2. RadA DNA binding and strand invasion activities.

(A) ssDNA binding assay. 25 nM labeled ssDNA (93 nt) were incubated with a range of RadA concentration (0, 0.4, 0.8, 1.6, 3.2, 6.4 µM) in 20 mM Tris-HCl, pH 8.0, 50 µg/mL BSA, 2 mM DTT and 0.5% Triton at 65°C for 10 min. Products were separated on a 0.75% native agarose gel and revealed by fluorescence. (B) Plasmid pUC19 (2686 bp) before and after purification of the supercoiled form. Samples were loaded onto an 1% agarose gel and compared to the supercoiled DNA ladder (N0472S, NEB). (C) D-loop formation assay with RadA. The gel from Figure 1 was stained with Sybr gold and imaged for the two following signals: green= labeled 93 nt ssDNA, red = dsDNA. The superimposition of both signals revealed in yellow the position of the primer engaged in D-loop. (D) D-loop formation assay at different temperature. 25 nM labeled ssDNA were incubated with 1.6 µM RadA during 10 min at 65, 70 or 75°C. Then 25 nM of supercoiled pUC19 were added and incubated for another 10 min. DNA products were separated on a 1.2% native agarose gel and revealed by fluorescence.

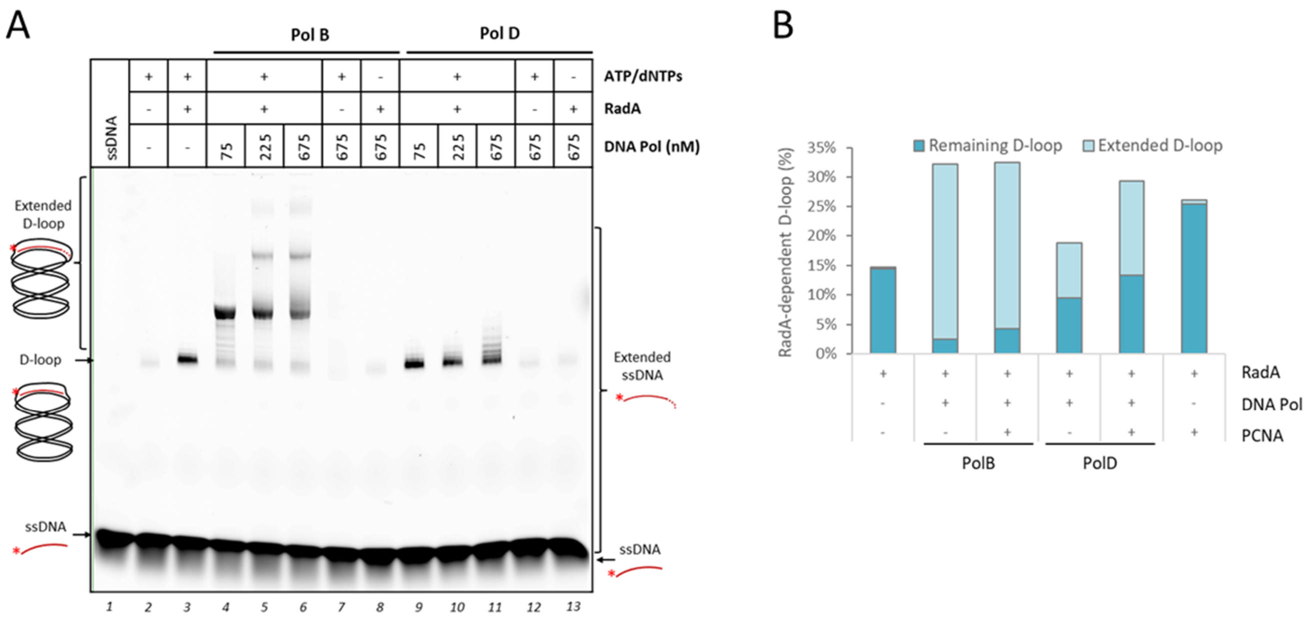

Figure S3. D-loop extension assays.

(A) D-loop formation and extension assays with increased concentration of Pol B and Pol D. 25 nM labeled ssDNA were first incubated with 1.6 µM RadA during 10 min at 65°C. Then 25 nM of supercoiled pUC19 were added and incubated for another 10 min. D-loop provided by RadA strand exchange activity is extended by (75, 225 or 675 nM) of Pol B or Pol D during 60 min at 65°C. DNA products were separated on a 1.2% native agarose gel and revealed by fluorescence. (B) Histogram representation of the D-loop extension assays as observed in Figure 2.B. RadA dependent D-loop (%), densitometry measurement of remaining D-loop (blue) or extended D-loop (light blue) as a percentage of total lane densitometry after data normalization and D-loop background subtracted.

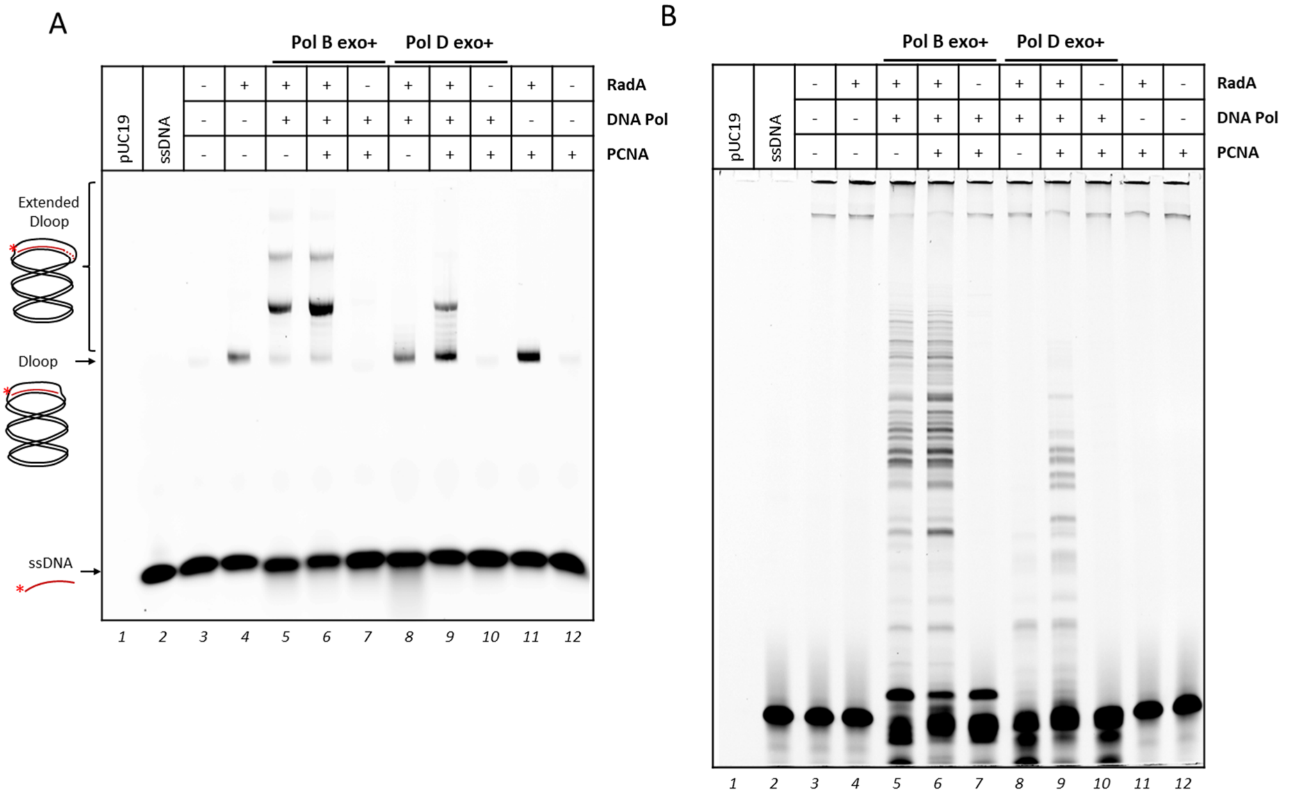

Figure S4. D-loop formation and extension assays with Pol B and Pol D exo+.
25 nM labeled ssDNA were first incubated with 1.6 µM RadA during 10 min at 65°C. Then 25 nM of purified scpUC19 were added and incubated for another 10 min. D-loop generated by RadA strand exchange activity is extended by 675 nM of Pol B or D exo+ (active for exonuclease activity) during 60 min at 65°C. DNA products were separated on a 1.2% native agarose gel (A) or 15% denaturing acrylamide gel (B). When indicated, 675 nM of PCNA were added together with DNA Pols. Labeled DNA products were revealed by fluorescence.

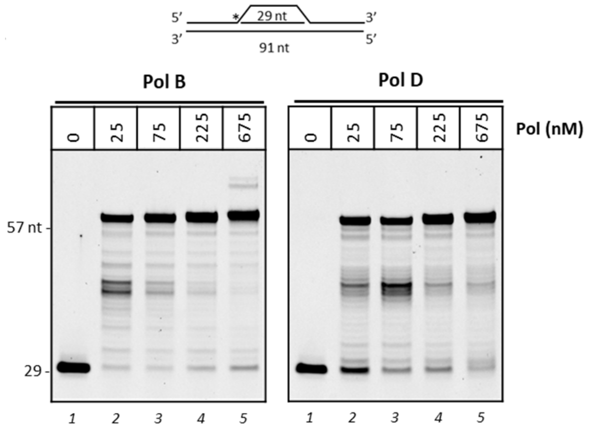

Figure S5. DNA synthesis activity on linear D-loop substrate with increased concentrations of Pol B and Pol D.
25 nM synthetic linear D-loop substrate were incubated with a range of concentration of PolB or PolD at 65°C for 60 min. DNA products were separated by gel electrophoresis onto a 15% denaturing acrylamide gel and revealed by fluorescence.

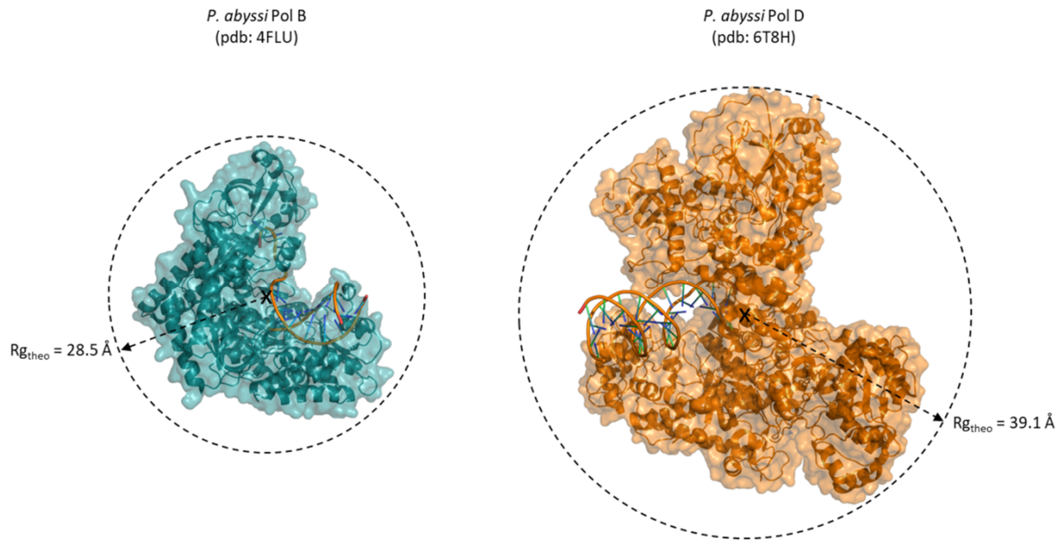

Figure S6. *P. abyssi* DNA polymerase structures.
On the left in blue, *Pa*Pol B structure (pdb:4FLU) and on the right *Pa*Pol D structure (pdb: 6T8H) within a DNA primer:template substrate inside. The predicted Rg was calculated from the structural data (without DNA chains) using the program CRYSOL and displayed with PYMOL software.

SUPPLEMENTAL TABLES

Table S1. *P. abyssi radA* DNA sequence optimized for *E. coli* expression.
In red, nucleotides modified compared to the original sequence.

ATGGGCAAAAAGAGCAGCGATCCGGCAGTTGTGGAAATTAATGATGTTGATGAACTGGAACTGGAAGTTGGTGAAGAAGTGACCTCGAAAAAGAAA
AAGAAAGAAAAAGAGATCCGCACCATTGAAGATCTGCCAGGTGTTGGTCCGGCAACAGCAGAAAAACTGCGTGAAGCAGGTTTCGATACCCTGGAA
GCAATTGCAGTGGCAAGCCCGATTGAACTGAAAGAAGTTGCAGGCATTAGCGAAGGTGCAGCACTGAAAATTATTCAGGCAGCACGTAAAGCAGCA
AATCTGGGTACCTTCATGCGTGCAGATGAATATCTGAAAAAGCGTGAAAGCATTGGTCGTATTAGCACCGGAAGCAAAAGCCTGGATAAACTGCTGG
GTGGTGGTATTGAAACCCAGGCGATTACAGAAGTGTTCGGTGAATTTGGTAGCGGTAAAACCCAGCTGGCACACACACTGGCCGTGATGGTTCAGCT
GCCGCCGGAAGAAGGTGGTCTGAATGGTAGCGTGATTTGGATTGATACAGAGAATACCTTTCGTCCGGAACGTATTCGCGAAATTGCTAAAAATCGT
GGTCTGGATCCGGATGAAGTTCTGAAACATATTTATGTGGCACGTGCATTTAATAGCAATCACCAGATGCTGCTGGTTCAGCAGGCAGAAGATAAAAT
TAAAGAGCTGCTGAATACCGATAAACCGGTTAAACTGCTGATTGTTGATTCACTGACCAGCCATTTTCGTAGCGAGTATATCGGTCGCGGTGCTCTGG
CAGAACGTCAGCAGAAACTGGCAAAACACCTGGCAGATCTGCATCGTCTGGCAAATCTGTATGAAATTGCCGTTTTCGTGACCAATCAGGTTCAGGCA
CGTCCGGATGCTTTTTTTGGTGATCCTACCCGTCCGATTGGAGGTCATATTCTGGCCCATTCAGCAACTCTGCGCGTTTATCTGCGTAAAGGTAAAGGT
GGCAAACGTGTTGCACGTCTGATTGATGCACCTCATCTGCCGGAAGGCGAAGCAGTTTTTCGTATTACCGAAAAAGGTATTGAAGATTAA

Table S2. Sequences of synthetic oligonucleotides used for linear D-loop extension assays.

Name	Sequence 5' to 3'	$S_{29/91}$	$S_{91/29/91}$
Up1_91 nt	GCCAGGGACGGGGTGAACCTGCAGGTGGGC GGCTGCTCATCGTAGGTTAGTATCGACCTATT GGTAGAATTCGGCAGCGTCATGCGACGGC	/	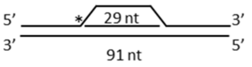
Up2_29 nt 5'Fam	AAGATGTCCTAGCAAGGCACCCTAGTAGC	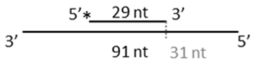	
Down_91 nt	GCCGTCGCATGACGCTGCCGAATTCTACCACG CTACTAGGGTGCCTTGCTAGGACATCTTTGCC CACCTGCAGGTTCACCCCGTCCCTGGC		
Trap_60 nt	AAGATGTCCTAGCAAGGCACCCTAGTAGCGT GGTAGAATTCGGCAGCGTCATGCGACGGC	/	/

Table S3. Sequences of synthetic oligonucleotides used for strand displacement assays.

Name	Sequence 5' to 3'	$S_{30/87/30}$
Up1_30 nt 5'Cy5	TGCCAAGCTTGCATGCCTGCAGGTCGACTC	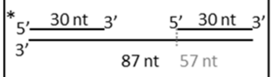
Down_87nt	CAGGAAACAGCTATGACCATGATTACGAATTC GAGCTCGGTACCCGGGGATCCTCTAGAGTCG ACCTGCAGGCATGCAAGCTTGGCA	
Up2_30 nt	ATTCGTAATCATGGTCATAGCTGTTTCCTG	
Trap_87 nt	TGCCAAGCTTGCATGCCTGCAGGTCGACTCTA GAGGATCCCCGGGTACCGAGCTCGAATTCGT AATCATGGTCATAGCTGTTTCCTG	/
